# Impact of non-proteinogenic amino acids in the discovery and development of peptide therapeutics

**DOI:** 10.1007/s00726-020-02890-9

**Published:** 2020-09-18

**Authors:** Yun Ding, Joey Paolo Ting, Jinsha Liu, Shams Al-Azzam, Priyanka Pandya, Sepideh Afshar

**Affiliations:** 1 Protein Engineering, Lilly Biotechnology Center, Eli Lilly and Company, San Diego, CA 92121 USA; 2 Professional Scientific Services, Eurofins Lancaster Laboratories, Lancaster, PA 17605 USA

**Keywords:** Non-proteinogenic amino acid, Peptide therapeutic, Discovery, Development

## Abstract

With the development of modern chemistry and biology, non-proteinogenic amino acids (NPAAs) have become a powerful tool for developing peptide-based drug candidates. Drug-like properties of peptidic medicines, due to the smaller size and simpler structure compared to large proteins, can be changed fundamentally by introducing NPAAs in its sequence. While peptides composed of natural amino acids can be used as drug candidates, the majority have shown to be less stable in biological conditions. The impact of NPAA incorporation can be extremely beneficial in improving the stability, potency, permeability, and bioavailability of peptide-based therapies. Conversely, undesired effects such as toxicity or immunogenicity should also be considered. The impact of NPAAs in the development of peptide-based therapeutics is reviewed in this article. Further, numerous examples of peptides containing NPAAs are presented to highlight the ongoing development in peptide-based therapeutics.

## Introduction

Peptides have drawn much attention in the drug discovery space. Since the first insulin native peptide was isolated and used for treating diabetes in the 1920s, over 150 peptide therapies have entered clinical studies and over 60 have been approved (Lau and Dunn 2018). Peptides are attractive drug candidates because of their high selectivity, low toxicity, and relative ease of synthesis. The structural diversity of the peptides is driven by proteinogenic and non-proteinogenic building blocks. Non-proteinogenic amino acids (NPAAs) are not naturally encoded in the human genetic code or found in the polypeptide chains. On the other hand, in organisms such as bacteria, fungi, plants, and marines, NPAAs are essential building blocks of polypeptide chains. Numerous NPAAs found in nature are analogs of natural amino acids (NAAs, or proteinogenic amino acids) (Fichtner et al. 2017) and some exist as secondary metabolites in many organisms.

While bioactive native peptides can intrinsically be used as drug candidates, their low bioavailability and short circulating plasma half-life hinder their direct use as therapeutics and often require structural optimization. NPAAs provide a toolbox of physiochemical properties that expand from NAAs (Stevenazzi et al. 2014; Xue et al. 2018). Various types of chemical and enzymatic synthesis methodologies have made NPAAs widely available (Stevenazzi et al. 2014a; Xue et al. 2018). Moreover, chemical and biosynthetic strategies such as orthogonal synthetase-tRNA approach and in vitro translation methods by mRNA display have enabled the construction of diverse peptide libraries that comprise NAA and NPAAs. These libraries have effectively been employed for target screening and selection to identify specific peptides (Kent 1988; Tian et al. 2004; Ma and Hartman 2012). A few examples of this approach will be highlighted here.

This review will focus on the roles of NPAAs in modulating stability, potency, permeability, oral bioavailability, and immunogenicity in peptides. We will present the predicament faced in the oral peptide therapeutic space and how NPAA incorporation can play a role in improving peptide–drug pharmacokinetic properties. While there is no magic combination of NPAA and NAA that can address challenges associated with peptide drug discovery, this review will provide numerous examples that validate the use of NPAA as a powerful tool to design stable, active, selective, and potent peptide therapeutics.

## Naturally occurring and synthetic NPAAs

Bacteria and fungi utilize non-ribosomal peptide synthetases (NRPSs) to synthesize hundreds of non-proteinogenic amino acids for incorporation in non-ribosomal peptides (NRP). These bioactive peptides have revolutionized the peptide drug space. Many natural and synthetically optimized NRPs have made it to the clinic, including penicillin precursor ACV-tripeptide, immunosuppressant drug cyclosporine, antibiotic vancomycin and many others (Saito et al. 1994; Offenzeller et al. 1996; Byford et al. 1997; Mootz and Marahiel 1997; van Wageningen et al. 1998; Keating et al. 2001). Here, we summarize different types of naturally occurring and synthetically made non-proteinogenic amino acids.

Natural amino acids, except for Gly, exists in two enantiomer configurations: l and d. The l enantiomers of amino acids are the dominant form in nature. In fact, some organisms have completely excluded d-amino acids to support peptide homochirality. On the other hand, many prokaryotic and eukaryotic organisms utilize d-amino acids in free form or in the context of polypeptide chains to induce a certain biological function. In Gram-positive eubacteria, d-Ala and d-Gln are produced in high quantities in the peptidoglycan layer of the cell wall to provide resistance from proteolysis (Hancock 1960). Toxic peptides, such as conotoxins isolated from carnivorous marine gastropod mollusks venom, contain d-Trp or d-Leu to block neuromuscular transmission in mammals (Jimenez et al. 2001). The toxic peptide, agatoxins, isolated form North American funnel-web spider *Agelenopsis aperta* showed compromised potency as a venom when d-Ser was substituted with Ser (Kuwada et al. 1994; Jimenez et al. 2001). d-Phe in gramicidin S and polymyxin B contributes to peptides’ antimicrobial efficacy in membrane disruption (Falagas et al. 2006). In humans and rodents, free d-Ser and d-Asp are distributed at high concentrations in different parts of the brain throughout embryotic development and postnatal life. d-Ser and d-Asp selectively potentiates *N*-methyl-d-aspartate (NMDA)-type excitatory amino acid receptor at its Glycine site to mediate neurotransmission (Hashimoto and Oka 1997). d-Asp is also found in elderly people in various tissues such as tooth, bone, brain, and eye lenses (Fujii 2002). d-Ser has been detected in β amyloid proteins of Alzheimer’s patients (Kaneko et al. 1995).

Post-translation modification (PTM) of proteins and peptides improves their functional and biological diversity beyond the 20 natural amino acids. There are over 20 different types of modifications to the natural amino acids. The PTMs such as phosphorylation, acetylation, and disulfide formation can be reversible and are commonly associated with signaling and metabolic processes. Irreversible PTM reactions are typically associated with physiological cascade processes such as blood coagulation, peptide bond cleavage, and protein splicing. Methylation is the most common PTM of Lys; however, some Lys residues in transcription factors are modified by ubiquitination, sumoylation, and acetylation to regulate target gene expression in response to extracellular signals. On a further note, modification of a Lys residue can influence the modification of the neighboring residues. Sumoylation of Lys in human and yeast may have a protective functionality against proteolysis (Freiman and Tjian 2003). For example, sumoylation of the Lys21 in IκBα prevents its ubiquitination and increases protein resistance to proteasome-mediated degradation (Desterro et al. 1998). Phosphorylation in prokaryotic and eukaryotic organisms is catalyzed by various kinases, where a phosphate group is transferred into the hydroxyl-containing amino acid. Phosphorylation is a common modification of Ser, Thr, and Tyr to mediate receptor activation and cellular transduction signaling. Another essential modification occurs through disulfide bond formation between two free thiol (SH) of cysteines within the same polypeptide chain or with other moieties. Cyclization occurs to increase the structural and enzymatic stability (Chung et al. 2013; Góngora-Benítez et al. 2014).

**Fig. 1:**
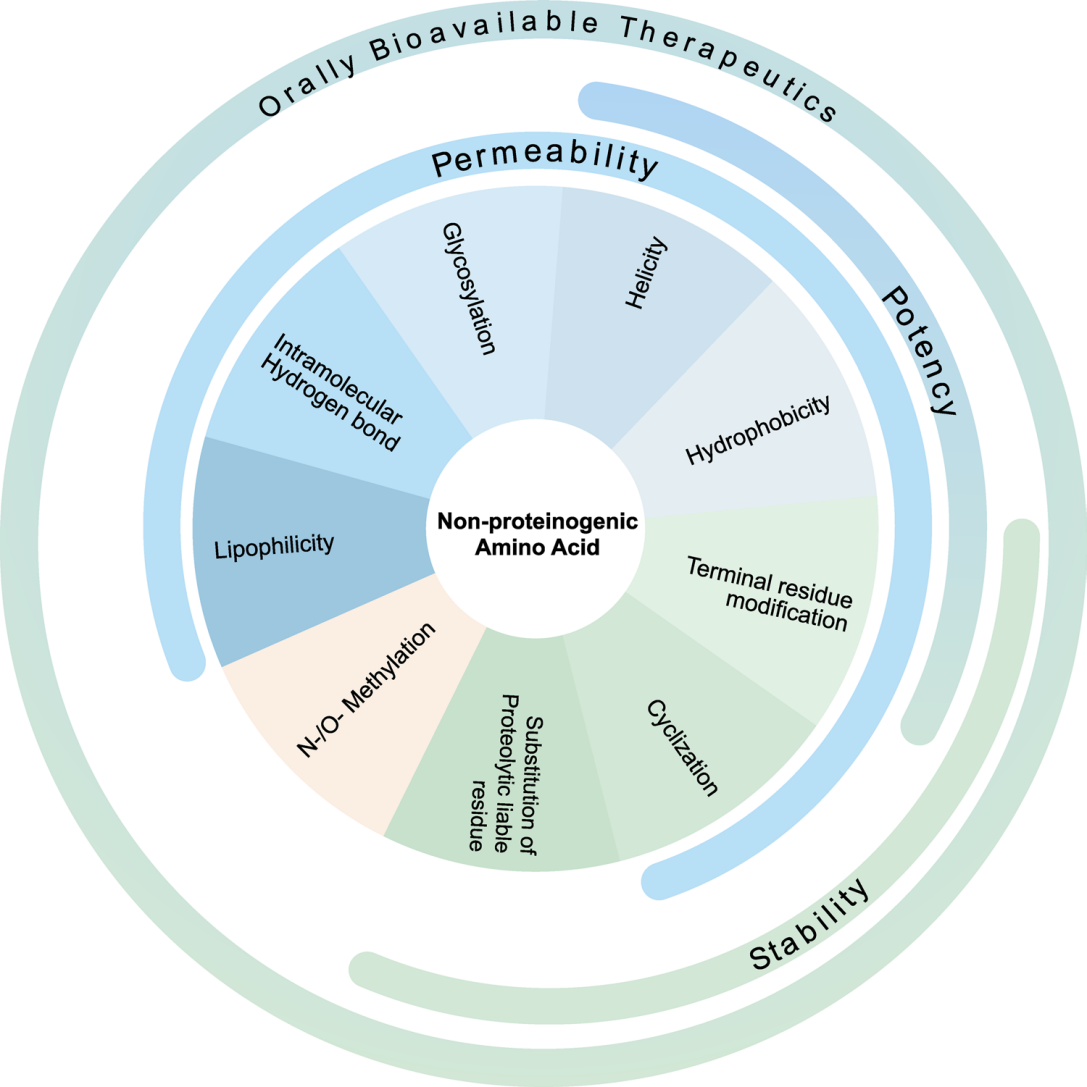
Different strategies of non-proteogenic amino acids incorporation to improve the pharmacokinetic properties of peptide drugs. The wide array of available NPAAs can be introduced in the peptide therapeutics to increase stability, potency, and permeability, which can lead to improved oral bioavailability

Synthetic peptides can be chemically modified internally, or at their C- or N-terminus to introduce stability, bioactivity, permeability, and bioavailability (Fig. 1). Acetylation of the N-terminus of short peptides has shown to improve peptidase stability in serum and hence their half-life (Wallace 1992). Protein glycosylation can increase protein–protein interaction and protein stability. Interestingly, glycosylation of peptides is suggested to improve peptide permeability, increase metabolic stability, and lower clearance rate, thereby improving bioavailability (Sola and Griebenow 2009; Moradi et al. 2016). A pioneering example is glycosylated analogs of the oral peptide therapy somatostatin. The glycosylated version retained its original activity and showed ten times higher bioavailability (Albert et al. 1993). Peptide half-life is expanded through lipidation, where long-chain saturated lipid is acylated to an amino acid to facilitate its binding to a carrier serum protein as demonstrated in the glucagon-like peptide-1 agonist to treat diabetes (Knudsen et al. 2000).

**Fig. 2:**
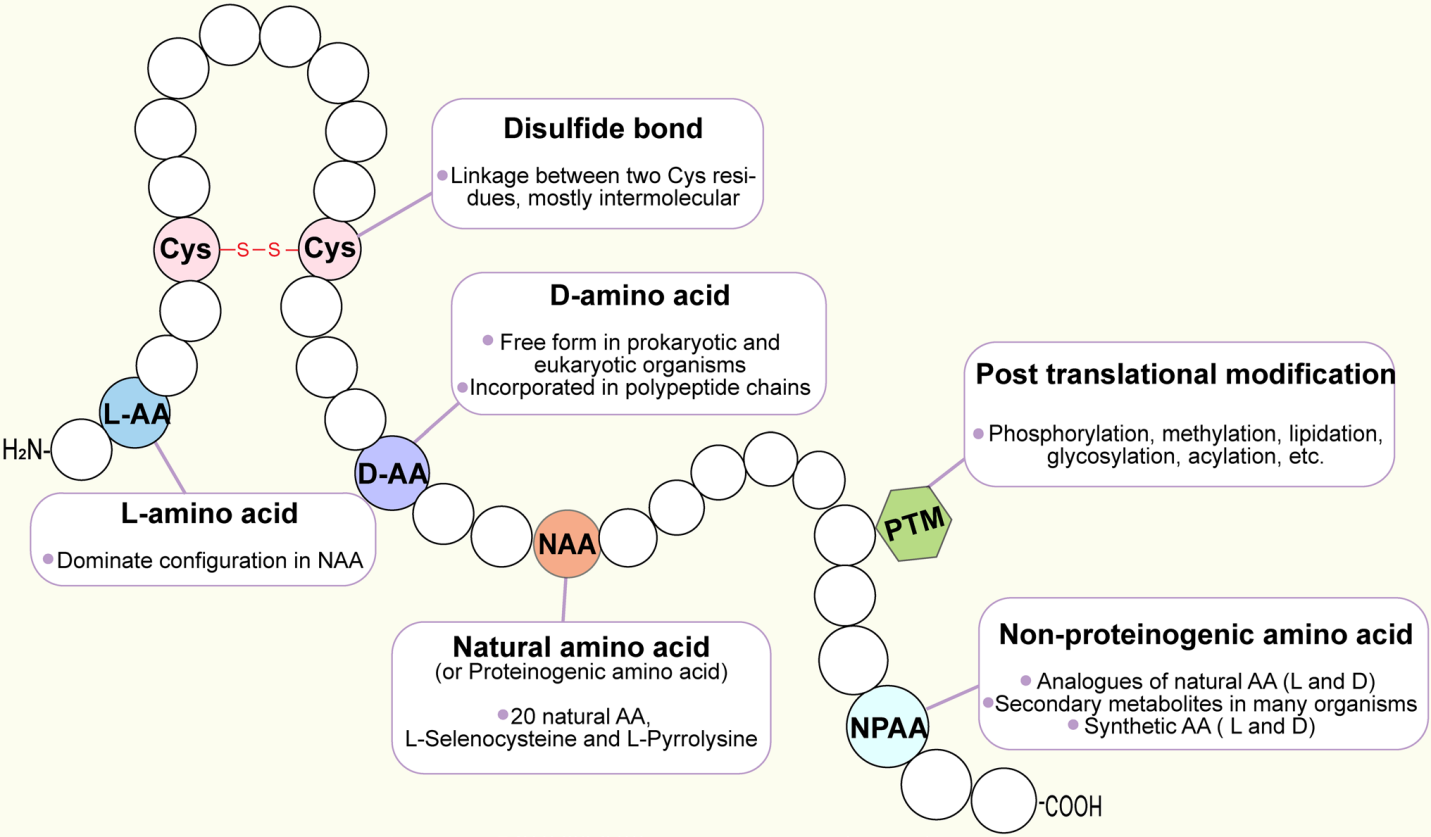
Peptide building blocks and modifications. Different building blocks such as NAA and NPAA with an l or d configuration can be incorporated into the peptide chain. Some of these building blocks can be further modified using a variety of post-translational modifications

There are over 800 naturally occurring NPAAs discovered and thousands of NPAA synthesized (Narancic et al. 2019). Most synthetic NPAAs are designed based on the natural amino acids and can be synthesized through chemical and biocatalytic processes, or by combination of both. The chemical route includes alkylations by Glyequivalents, amination by tandem reactions, reduction, or alkylation, carboxylation, cyanation combined with hydrolysis, as well as other side chain modifications (Agostini et al. 2017). The challenges for chemical synthesis of NPAA include stereoselectivity and the low production yield. The biocatalytic route utilizes various enzymatic reactions to produce enantiomeric NPAAs with a higher yield, it requires fewer steps compared to chemical synthesis, and often can be done in the aqueous media. The major limiting factor for this method is the high cost of the cofactors when large-scale production is required (Narancic et al. 2019). Overall, artificial NPAAs including D enantiomer, PTM, and analogous amino acids serve as versatile tools in various scientific disciplines, including drug discovery, study of protein structure, protein trafficking, and protein optimization (Narancic et al. 2019). They are key building blocks for introducing desired functions and properties in synthetic peptide drugs (Fig. 2).

## NPAAs incorporation for improving peptide stability and related properties

Despite lack of an established guideline, several reviews outline different strategies and various NPAA usage to enhance peptide stability (Gentilucci et al. 2010; Cavaco et al. 2017; Stone and Deber 2017). These strategies include substitution of proteolytic liable residues with NPAA, terminal residue modifications, and peptide cyclization.

Incorporation of NPAAs can prevent proteolysis of peptides through stabilization of backbone conformation and/or by elimination of the enzyme recognition site (Gentilucci et al. 2010). One example cited is anti-angiogenic heptapeptide (DRVYIHP), which is degraded by angiotensin-converting enzyme (ACE) and dipeptidyl peptidase 3 (DPP-3). DPP-3 and ACE cleave the peptide bond at two sites, Asp1–Arg2 and His6–Pro7, respectively. Replacement of Val3, Ile5, or His6 with the rigid NPAA, *N*-(9-fluorenylmethoxycarbonyl)-*cis*-3-(aminomethyl) cyclobutanecarboxylic acid (ACCA), resulted in proteolytic resistance against ACE and DPP-3 (Wester et al. 2017). Similarly, substitution of Lys1 in the membrane-active peptide (MP: KKVVFKVKFKK) (Hong et al. 1999) with the bulky and positively charged 3-[2′-(*tert*-butyloxycarbonyl)-hydrazino (Jawa et al. 2013) improved its proteolytic stability (Oh and Lee 1999). Another example is the human glucagon-like peptide (GLP-1), a natural hormone that plays a key role in lowering blood sugar by stimulating insulin secretion. Dipeptidyl peptidase 4 (DPP-4) cleaves the His7–Ala8 peptide bond and results in GLP-1 inactivation. In taspoglutide, an analog of GLP-1, 2-aminoisobutyric acid (Aib) substitution at positions 8 and 35 prevented proteolytic cleavage by DDP-4, plasmin, and plasma kallikrein (Sebokova et al. 2010; Dong et al. 2011). Like GLP-1, GLP-2 is susceptible to proteolysis by DPP-4. To prevent degradation, native GLP-2 was modified with Gly2 and Norleucine10 (compound 2). Structural–activity relationship was used to further optimize compound 2 by substitutions with D-Phe11 and Leu16 and C-terminal amidation to generate apraglutide (FE 203799), a highly selective and potent GLP-2 receptor agonist (Wisniewski et al. 2016; Suzuki et al. 2020). Incorporation of hydrophobic residues, D-Phe11 and Leu16, reduced metabolic clearance in rat from 9.9 mL/min/kg (compound 2) to less than 0.3 mL/min/kg. Improved metabolic clearance rate was correlated with much enhanced plasma protein binding due to increased hydrophobicity (Wisniewski et al. 2016). Semaglutide (Ozempic^®^, Novo Nordisk) is a stable GLP-1 (7–36) analog in which Ala8 is replaced by Aib (Lau et al. 2015; van Witteloostuijn et al. 2016). Furthermore, incorporation of an 18-carbon diacid acyl chain in semaglutide resulted in its binding to albumin, improving its serum stability and half-life. The C18 chain was conjugated to Lys26 via a γGlu-2X 8-amino-3,6-dioxaoctanoic acid (OEG) linker. Consequently, half-life of semaglutide was extended to 160 h in human plasma (Lau et al. 2015, van Witteloostuijn et al. 2016), a significant improvement compared to the half-life of GLP-1 (1.5–5 min). In liraglutide (Victoza^®^, Novo Nordisk), conjugation of a 16-carbon saturated fatty acid (palmitic acid) to Lys26 of GLP-1 through a gamma glutamate (γGlu) linker (Lorenz et al. 2013; Aroda 2018; Knudsen and Lau 2019; Lear et al. 2019) extended peptide half-life up to 15 h (Agerso et al. 2002; Hui et al. 2002; Knudsen and Lau 2019). Addition of a C16 chain or a 12-aminododecanoic acid to Asp34 of GLP-1 demonstrated an improved blood retention in rat. In in vitro studies, it was shown that incorporation of C16 in GLP-1 results in increased proteolysis resistance to DPP-4 compared to C12 and C8 side chains (Li et al. 2015). Exendin-4 (Byetta^®^), a naturally occurring non-human peptidic agonist of GLP-1R, shares 50% homology with human GLP-1 (Göke et al. 1993; Underwood et al. 2010). Half-life of Exendin-4 was extended from 2.4 h (Bray 2006; Aroda 2018; Lear et al. 2019) to 8 days by fusing albumin to the C-terminal Lys40 (Lorenz et al. 2013). As a result, the drug is administered once a week compared to twice daily injections of the parent peptide. Enhanced stability and prolonged half-life achieved through inclusion of NPAAs that bind to albumin have been observed in peptides other than GLP-1 analogs. An anti-angiogenic peptide F56 (WHSDMEWWYLLG) was previously shown to bind to vascular endothelial growth factor receptor-1 and inhibit blood vessel formation in both zebrafish embryos and chicken chorioallantoic membrane (An et al. 2004). Conjugation of maleimidopropionic acid (MPA) to the N-terminal of F56 resulted in covalent interaction of the peptide with Cys34 of albumin. Consequently, peptide half-life was increased from 0.4249 to 6.967 h in rats (Feng et al. 2018). Similarly, attachment of a palmitic acid (C16) chain to C-terminus of an anti-human immunodeficiency virus-1 (HIV-1) peptide YIK (EMTWEEWEKKIEEYIKKIEEILKKSQNQQLDL) extended its serum half-life from 1.3 to 5.9 h in mice (Su et al. 2019).

Cyclization of peptides using natural or NPAAs can increase conformational stability and minimize protease susceptibility. Different approaches, such as head–tail, side chain–side chain, head–side chain or side chain–tail are employed for peptide cyclization (Katsara et al. 2006; Frost et al. 2015, 2016; Chow et al. 2019). The effect of head–tail cyclization in the stability of a linear peptide that corresponds to 279–287 sequence of glycoprotein-D-1 (gD-1, LLEDPVGTVA) of herpes simplex virus (HSV) was investigated by three different methods: peptidic bond between N- and C-terminus, disulfide bond using flanking cysteines, and thioether linkage using *N*-acetyl-leucine and C-terminal cysteine (Tugyi et al. 2005). Cyclic peptides resisted enzymatic hydrolysis in the presence of lysosomal fraction at pH 3.5 and 5.0 for the duration of the assays at 180 min. It should be noted that only 8 and 33% of the linear parental peptide remained intact in the presence of lysosomal preparations at pH 3.5 and 5.0, respectively. Cyclized gD-1 peptides also showed significant stability in the presence of 10 and 50% human serum; however, only the gD-1 with thioether linkage remained fully intact. The linear 11-mer peptide is of special interest for eliciting immune response against the virus since it maps to the HSV-1 epitope for neutralizing antibodies. Although the effect of gD-1 cyclization on antibody response is yet to be determined, the studies suggest that head–tail cyclization of the linear peptide, especially in the form of thioether linkage, can result in peptide stability.

In a similar study, the stability and potency of linear and macrocyclic analogs of compound I, a phosphino dipeptide (PDP) isostere inhibitor of β-secretase (BACE1), were compared (Huber et al. 2009). Different side chain–side chain modifications were introduced to linear compound I to generate cyclized analogs (compounds II-P1 to II-P4). Cyclized compound II-P1 was the most stable in the presence of human serum. After 120 min, compound I was completely degraded, whereas 20% of the cyclic compound II-P1 remained intact at 160 min. Similarly, half-life of compound II-P1 was increased (43.9 min) compared to its linear parent, compound I (14.8 min). It should be noted that as peptide stability was improved, its potency was decreased. IC50 of the compound II-P1 was determined to be 47 nM, fourfold weaker than the linear compound I (12 nM). This suggested that improvements in the peptide stability might come at the expense of its other druggable attributes. Therefore, stability campaigns should not be carried out unidirectionally and they should be considered in the context of other peptide properties, such as potency.

Macrocyclic peptides that disrupt and inhibit amyloid-β (Aβ) peptide aggregation were designed and analyzed for therapy against Alzheimer’s disease (AD) (Kalita 2020). Seven analogs were tested. All variants were tail to side chain cyclized (SP1 through SP6) except for the control linear peptide (LP1). SP1–SP3 analogs contained NPAAs *N*-methyl adipic acid, *N*-methyl glutaric acid, and *N*-methyl succinic acid, respectively, that were stapled to Lys residue for cyclization. SP4 to SP6 were derived from SP1 to SP3 and contained the turn inducing NPAA, anthranilic acid (Ant) to restrict peptide flexibility. SP1 through SP6 showed inhibition of amyloid aggregation in vitro in the time-dependent thioflavin T fluorescence assay. SP1 through SP6 also showed disruption of Aβ peptide aggregates when added to the preformed amyloid fibrillar post-48 h of assembly. SP2 and SP5 that contained *N*-methyl glutaric acid with and without Ant, respectively, showed the best efficacy. The proteolytic stability of all peptides were tested in culture media containing 10% FBS and peptide retention was measured through RP-HPLC and MALDI-TOF. SP1 through SP6 remained fully intact up to 25 h, whereas the linear LP1 degraded after 1 h.

Although the guidelines as how to best combine natural and non-proteinogenic amino acids to generate proteolytically stable peptides (Liang et al. 2013) are yet to be determined, key substitutions by NPAA have proven to be an effective way to enhance peptide proteolytic resistance, structural stability, and plasma half-life.

## NPAAs incorporation for increasing peptide potency

Peptides containing NPAAs can be designed to modulate protein–protein interaction. In a computational approach, different motifs that come in contact with the matrix metalloproteinase (MMP) were used to design enzyme inhibitors. The inhibitors contained NPAAs that mimic side chains of different motifs with the highest predicated MMP binding value. Examples of these UNAA are cyclohexylglycine (CHG), homoserine (HSER) and homophenylalanine (HPE), and 3-cyclopentyl-alanine (CPA3). Analysis of more than 4000 motifs identified DI-F/Y/K and HSER-GF as potent inhibitors of MMP-2 and CHG-I/V-L/Q/M/I/L as potent binders of MMP-7 (Gfeller et al. 2013; Song et al. 2018). Compstatin (ICVVQDWGHHRCT) is a cyclic inhibitor of complement C3 with the aromatic ring structures at positions 4 and 7 that appear to be essential for its activity. Incorporation of 1-methyltryptophan at position 4 resulted in 264 times higher activity than the parent peptide due to the increased hydrophobicity. In contrast, a polar residue at position 7 is much preferred for C3 binding (Katragadda et al. 2006). In another example, a set of bulky and hydrophobic NPAAs was used to replace Ala1 of the human leukocyte antigen HLA-DQ blocking peptide (ADAYDYESEELFAA). A previous X-ray structural study indicated that the peptide did not fully occupy the binding pocket of HLA-DQ and a hydrogen-bond network between Glu and His residues of HLA-DQ was located at the bottom of the pocket. Among the many bulky and hydrophobic NPAAs tested, pyroglutamate (Pyr)–Ala substitution improved the binding affinity by fivefold in an in vitro assay (Kim et al. 2004; Kapoerchan et al. 2010).

HIV-1 infection is initiated by the binding of the virus surface protein gp120 to CD4 of the host T cell (Weiss et al. 1990; Harris et al. 2011) and is facilitated by the co-receptors CCR5 and CXCR4 on the immune cells (Chen 2019). A selection of a phage display library against gp120 resulted in the discovery of a 12-residue long peptide (12p1: RINNIPWSEAMM) with blocking activity (Ferrer and Harrison 1999). The side chain of Trp7 was shown to play a pivotal role for contacting gp120. Hence, the adjacent residue Pro6 was subjected to a full spectrum analog scanning. As a result, peptides with 4-azidoproline substitutions with improved affinity were identified. Both *cis* and *trans* versions of 4-azidoproline were synthesized and tested in binding and cell-based assays. Although both peptides increased the hydrophobic patch around Trp7, the peptide containing *cis*-4-azidoproline exhibited tenfold stronger binding affinity and blocking activity compared to the parent peptide (Gopi et al. 2006, 2008, 2009). Binding kinetics study and cell infection assay were used to identify NNIPW as the core motif for binding and bioactivity. To rescue the potency loss due to peptide minimization, a set of natural and NPAAs (Arg, Glu, citrulline (Cit: (2S)-2-amino-5-(carbamoylamino)pentanoic acid), Lys and Phe) was used to replace N-terminal Arg-Ile. Cit was selected based on the highest binding affinity and biological activity. Since the activity of the new analog was still lower than the parent peptide, two NPAA Pro variants, FtP (ferrocenyltriazole-Pro) and L-Bta (L-3-benzothienylalanine), were designed to replace Pro6 and Trp7. The resulting peptide (Cit-NNI-FtP-Bta-S) showed a comparable level of activity as full-length parent peptide. No further data were disclosed to compare the potency of the resulting peptide to the previous peptide containing *cis*-4-azidoproline (Umashankara et al. 2010; Kamanna et al. 2013).

A similar approach has been exercised for the discovery of truncated incretin analogs. Bristol-Myers Squibb discovered an 11 residue GLP-1(7–15) analog with Aib at the position eight to confer DPP-4 resistance. Over 50 NPAAs were tested and only a few key NPAAs substitutions resulted in increased peptide potency. These included l-α-methylphenylalanine (2-F) at position 12, l-biphenylalanine (4′-OMe, 2′-Et) at position 15, and biphenylalanine (4′-OMe, 2′-Et) and homohomophenylalanine at the C-terminus of the peptide. The resultant peptide exhibited comparable activity to the naïve ligand in mouse and human cAMP assays (Mapelli et al. 2009; Haque et al. 2010a, b).

HTLV-I protease (PR) plays an essential role in regulating the replication of human T cell lymphotropic virus type 1 (HTLV-1) (Hatanaka and Nam 1989), which is a close relative of HIV (Quaresma et al. 2015; Khan et al. 2017). A series of inhibiting peptides were designed based on the PR substrate sequence (PQVLPVMHP) (Nguyen et al. 2008). Initially, an active analog was identified by substitution of Leu4 and Pro5 with (2*S*,3*S*)-3-amino-2-hydroxy-4-phenylbutyric acid (allophenylnorstatine, Apns) and (*R*)-5,5-dimethyl-1,3-thiazolidine-4-carboxylic acid (Dmt), respectively (Maegawa et al. 2004). The truncated peptide (Ac-QV-Apns-Dmt-I-VM) maintained 66% HTLV-1 inhibition of the parent peptide. To rescue the loss of activity, various natural (Val, Ala, Leu, Ile, Phe, Gln, Thr and Met) and NPAAs (l-methylthioalanine (Mta), l-*tert*-leucine (Tle) and l-(+)-a-phenylglycine (Phg)) were incorporated at positions 1, 2, 5 and 6 of the truncated peptide. The resultant peptide (Ac–Phg–Tle–Apns–Dmt–Ile–Met) had comparable activity as the parent peptide. Replacement of Apns with its diastereomer did not result in an additional gain of activity (Kimura et al. 2007; Nguyen et al. 2008).

Day and colleagues revealed a unique strategy to generate GLP-1R and glucagon receptor co-agonists through the use of NPAAs. The 29-residue hybrid peptide (H-Aib-QGTFTSDYSKYLDEQAAKEFI-C(PEG)-WLMNT-NH2) containing the key residues of both native GLP-1 and glucagon was generated. To increase the glucagon activity, an intermolecular lactam bridge was incorporated between Glu16 and Lys20, to enforce its helical structure. In addition, a 40 kDa polyethylene glycol chain (PEG) was conjugated to Cys24 to enhance solubility at high concentrations (> 25 mg/ml) and to prolong plasma half-life. Administration of this peptide to mice with diet-induced obesity resulted in lowering blood glucose and significant weight loss compared to the control group (Day et al. 2009; Lorenz et al. 2013).

Fatty-acid conjugation is an efficient way to optimize the activity of antiviral peptides by enhancing their interaction with the host membrane. Infection by influenza (flu) is triggered by binding of the influenza type A virus membrane protein, hemagglutinin (HA) to the host sialylglycoconjugate receptors (Lorenz et al. 2013). HA is a homotrimeric lectin that is present at 600–1200 copies per virus. Phage display was used to identify a 15-residue long peptide (ARLPRTMVHPKPAQP) that bound to HA and interrupted its interaction with sialylglycoconjugate receptors. The peptide was matured by conjugating an 18-carbon (C18) stearoyl group to its N-terminal alanine, which resulted in the formation of peptide amphiphiles. The amphiphilic peptide comprised a hydrophilic single- or double-alkyl tail and a hydrophilic biological active domain. The alkyl tail resulted in peptide aggregation, nano-fibrillization, self-assembly, and liposome incorporation. Self-assembly and formation of micelles enhanced peptide inhibitory function through multivalency (Hartgerink et al. 2001, 2002; Matsubara et al. 2010; Missirlis et al. 2010; Chen and Zou 2019). A 16-carbon (C16) alkyl chain conjugation at the N-terminus of the same peptide was shown to have a similar effect (Hüttl et al. 2013; Skalickova et al. 2015). Similarly, conjugation of a C16 chain to the anti-HIV-1 peptide YIK, mentioned above, resulted in twofold improved potency. It was suggested that improved potency was due to enhanced binding of the peptides to the membrane of both host cells and viruses (Su et al. 2019). It is important to mention that an amphiphilic peptide may contain either a C- or N-terminal alkyl tail and the biological activity can be dramatically affected by the position of the alkylation. Another HIV-1 peptide inhibitor C34 (WMEWDREINNYTSLIHSLIEESQNQQEKNEQELLGSGC) was initially engineered with a C- (C34-Chol) or N-terminal (Chol-C34) cholesterol group. C34-Chol accumulated in cell membrane at a higher level and was 50-fold more active than the parental C34, which was 50-fold more active than the Chol-34 in single-cycle infectivity assay, suggesting that the C-terminal membrane anchor is more favorable for this peptide (Ingallinella et al. 2009).

## NPAAs incorporation to enhance peptide permeability

A few strategies to promote peptide membrane permeability including enhanced peptide helicity, lipophilicity, intermolecular hydrogen bond formation, as well as glycosylation are discussed next. NPAA incorporation is a well-established strategy to increase the permeability of the highly cationic antimicrobial peptides (AMPs). De novo 11-residue AMP (Ac-KA∆FWK∆FVK∆FVK-CONH_2_) was rationally designed to incorporate Lys, α, β-dihydrophenylalanine (∆F), and Trp at key positions to induce amphipathic α-helical structure for fast permeation through the bacterial membrane (Pathak and Chauhan 2011). In a separate study, the substitution of Lys by ornithine (Orn), (Cbf-14-2: RLLR-Orn-FFR-Orn-LKKSV-NH2) resulted in fourfold superior antimicrobial activity and increased protection in mice infected with penicillin-resistant *E. coli* compared to the parent peptide (RLLRKFFRKLKKSV). Increased antimicrobial activity of Cbf-14-2 was due to increased helicity of the peptide with enhanced membrane rupture as shown by transmission electron microscopy (Kang et al. 2017). Other modifications, including peptide truncation and incorporation of NPAA, have led to the generation of AMPs with reduced size (dipeptides and tripeptides derivatives) and high passive permeation efficiency (Strom et al. 2003; Haug et al. 2004; Svenson et al. 2009; Flaten et al. 2011).

It has been suggested that enhanced helicity and hydrophobicity of the constrained peptides promote cell permeability. Cellular permeability of octa-arginine analogs, composed of d- and l-arginine residues, were investigated in HEK cells and lipid vesicles model. The octa-arginine variants containing at least six adjacent arginine residues showed higher cytoplasmic and nuclear penetration (Purkayastha et al. 2013). It is speculated that α-helicity induced in peptides with six or more arginines is the key contributor to peptide–membrane interaction. Indeed, peptides with restrained α-helical conformation are shown to benefit cellular uptake due to increased stability of amphipathic structures upon membrane interaction. Side chain stapling of peptide residues was commonly employed to stabilize its α-helical structure several efforts were made to staple the helical peptides by introducing NPAA containing olefin-bearing tethers to enhance conformational stability and cell permeability. These peptides are typically constructed by inserting ring-closing metathesis of olefin-terminated (*S*)-2-(4-pentenyl)alanine or (*R*)-2-(7-octenyl)alanine into the helix (Walensky et al. 2004; Bernal et al. 2007; Moellering et al. 2009; Chang et al. 2013; Wang et al. 2014; Teng et al. 2016). BH3 domain contains a conserved α-helical segment derived from Bcl-2 family proteins (Gross et al. 1999) that are cell impermeable. Stapled variants of BID BH3 peptide (SAHB_A_: EDIIRNIARHLAQVGDS-Nle-DRSIW) were generated with hydrocarbon cross-linking at residues Gln13 and Ser17. Increased helicity, lipophilicity, and cellular permeability in cultured leukemia cells were observed with SAHB_A_. Intravenous administration of SAHB_A_ suppressed tumor growth in the immunodeficient mice bearing human leukemia xenografts (Walensky et al. 2004). A stapled version of Bim BH3 peptide (IWIAQELRRIGDEFNAYYARR) was generated by cross-linking of residues Arg9 and Glu13 by ruthenium-catalyzed olefin metathesis. The resultant peptide showed increased helicity and decreased protease susceptibility compared to the parental peptide. Furthermore, substitution of Trp2, Glu6, and Ala16 with cyclic β-amino acids such as aminocyclopropane-1-carboxylic acid (ACPC) and succinyl-aminocyclopentanecarboxylic acid (sAPC) resulted in 100-fold increase proteolytic stability, while maintaining permeability and potency of the parental peptides. It was suggested that stapling peptides might increase their tendency to aggregate, limiting use of this strategy (Checco et al. 2015). Furthermore, a recent study suggested that increased hydrophobicity and not the peptide helicity is the key driver of cellular uptake. In this study, the cellular uptake of FITC-labeled unstapled and staple peptides with different physicochemical properties, such as length and hydrophobicity, were evaluated by confocal microscopy and flow cytometry. The results showed higher cellular uptake with unstapled variants. Interestingly, no correlation was observed with permeability and helical content. Since elimination of ethylene form ruthenium-catalyzed olefin metathesis lessened peptide hydrophobicity in the stapled form, the authors argued that the increased hydrophobicity of the unstapled peptides resulted in increased interaction with membrane, which possibly led to the enhanced cellular uptake rate (Sakagami et al. 2018).

Inability of peptides to penetrate the blood–brain barrier (BBB) to gain access to the brain and central nervous system (CNS) is the key drawback for their clinical use in neurodegenerative diseases. Chemical modifications to increase the lipophilicity such as backbone stereochemistry and NPAA incorporation are used to promote passive cell permeation. For example, peptide variants of BBB shuttles (N-MePhe)_*n*_ exhibited higher passive permeability with increased lipophilicity. Transport efficacy of the stereoisomeric variants containing one to four residues with l- or d-MePhe were accessed across the cell membrane in the parallel artificial membrane permeability assay (PAMPA). Homochiral peptide Ac-(D-N-MePhe)4-CONH2 showed higher permeability compared to its homochiral l counterparts and heterochiral enantiomers. (N-MePhe)_*n*_ variants containing hydrophobic cyclohexylalanine (Cha: wider side chain) and 2-naphthylalanine (2Nal: longer side chain) at the N-terminus were conjugated to small neurodrugs including 3,4-dihydroxyl-phenylalanine (L-DOPA), 4-aminobutyric acid (GABA), or nipecotic acid. Peptide variants with Cha showed the highest shuttling performance in PAMPA assay. Furthermore, incorporation of the chlorinated variants of Phe in the dipeptide and tripeptide–neurodrug conjugates significantly increased their permeation in PAMPA, indicating increased lipophilicity enhances peptide ability to cross the cell membrane (Malakoutikhah et al. 2014).

Peptide-derived inhibitors of pain may represent viable non-opioid alternatives without the undesirable side effects of morphine. However, endogenous opioid neurotransmitters such as enkephalin (Enk, with Leu5 or Met5) and endomorphin (EM-1: YPWF) have been poor drug candidates because of their limited ability to cross the BBB after systemic administration (Egleton and Davis 2005). To increase the lipophilicity of Leu-Enk (YGGFL), an endogenous µ/δ opioid receptor agonist, NPAAs containing C8 or C12 lipoaminoacid side chain (2-amino-d,l-octanoic acid hydrochloride) and carboxyamide was introduced at N- and C-terminus respectively. Both lipidic derivatives (C8-Enk-NH2 and C12-Enk-NH2) showed enhanced permeability and stability in CaCo-2 cell monolayer compared to the parent peptide, with higher values reported for C8-Enk-NH2. Interestingly, N-terminal acetylation of Enk with C12 (Ac-C12-EnK-NH2) did not show permeability through CaCo-2 monolayers due to its poor solubility. With intranasal administration of C8-Enk-NH_2_ in rats, nanomolar concentration of the peptide was detected in the olfactory bulbs and in the brain using LC–MS/MS. Lower concentrations detected in blood was an indication of fast uptakes in CNS compared to the circulation, suggesting enhanced permeability through the olfactory epithelial pathways. The introduction of sugar moieties at N- or C-terminus of Enk and analogs have been studied to improve the cell uptake by glucose transporters. *β*-d-Glucosyl (Glc) or *β*-d-galactosyl (Gal) were added to the N-terminus of an Enk analog (Y^D^ MGFP) (Szekely et al. 1977; Rodriguez et al. 1990), and *O*-linked *β*-d-glucose (Glc) was introduced at the C-terminus of Met-Enk (YGGFM) (Polt et al. 1994). All glycol analogs showed significant antinociceptive activity in mice compared to the parent peptide. To promote the entry of EM-1 to CNS, three modifications were introduced: (1) N-terminal Tyr was modified by guanidino-addition, (2) Pro was substituted with d-Ala, and (3) C-terminal Phe was replaced by chloro-Phe. When the resultant variant (guanidino-Y^D^AW*p*^Cl^F-NH2) was administered subcutaneously in mice, significantly stronger analgesia with improved duration was observed (Liu et al. 2006). The C-terminal Phe in EM-1 was shown to be the key residue to influence binding affinity and selectivity (Janecka and Kruszynski 2005). When Phe was substituted with (thienyl)-α-methylene-β-amino acids (Map), a structurally constrained amino acid, the new peptide YPW-(thienyl)Map resulted in a fivefold increased binding affinity (sub-nanomolar) to the µ-opioid receptor expressing HEK293 cells. In addition, enhanced functional activity of the peptide was observed in mice indicating improved BBB permeability (tail-flick and formalin tests) (Liu et al. 2015). In a similar study, it was shown that the attachment of lactose to the N-terminus of the EM-1 peptide improved the metabolic stability by 20-fold in human plasma and resulted in 700-fold increase in membrane permeability in CaCo-2 cell monolayers (Varamini et al. 2012). The significant enhancement in cell permeability of the glycosylated EM-1 was possibly due to the transport through a lactose receptor and lactose selective transporter. Glycosylated EM-1 retained its binding affinity to the µ-opioid receptor. Oral administration of glycosylated EM-1 in the rat model of neuropathic pain resulted in significant increase in receptor agonism, indicating enhanced oral availability and BBB permeation.

Peptide-based inhibitors targeting oligomerization of Aβ hold significant promise for treatment of AD. The aromatic nature of the amyloid interface is a critical factor for its self-assembly. Hence, breaking β-sheets, the structure enriched in early intermediates of Aβ, can be a potential therapeutic for AD. For that purpose, in an approach that is referred to as C^α^-methylation β-breakage strategy, peptides containing α-methylated amino acids were considered. N-Methylation was incorporated to minimize the self-association of the peptide inhibitor. The dipeptide (NH_2_-^D^W-Aib-OH) with high water solubility was rationally designed to interact with early low-molecular weight species of Aβ. The peptide was shown to inhibit the growth of toxic globulomer assemblies in cultured PC12 cells. The ability of the dipeptide to cross the BBB was evaluated in CD-1 mice by either intravenous, oral, or intranasal administration, and the peptide levels were determined in both plasma and brain tissue by LC–MS/MS analysis. The authors reported great bioavailability upon oral (39%) and nasal (55%) application in mice. Moreover, administration of the peptide in the AD transgenic mice models overexpressing hAPP resulted in significant reduction of amyloid deposit and improved cognitive performance (Morris water maze test) (Frydman-Marom et al. 2009).

Peptide cyclization eliminates charged termini and may facilitate internal hydrogen bond formation, thereby increasing membrane permeability (Burton et al. 1996; Rezai et al. 2006b). Cyclosporin A (CsA), the gold standard permeable peptide, is a macrocycle with 7 N-methylated NPAAs (Augustijns et al. 2000). CsA has been extensively studied to understand what governs peptide permeability. One pertinent question was whether N-methylated NPAAs must be included in the peptide sequence to promote intramolecular hydrogen bonding that is deemed necessary for permeability. Cyclic hexapeptide diastereomers were identified based on molecular modeling of intramolecular hydrogen bond patterns (Watts and Forster 2012). The hexapeptides were designed based on the sequence cyclo (LLLLPY) and contained l- and d-residues. Variants were tested in PAMPA assay for passive diffusion at 72-h time point (Rezai et al. 2006b) and cyclo (^D^L^D^LL^D^LPY) was identified with the highest permeability (log *P*_E_ = − 6.2). The permeability of the linear version was determined to be below detection. CsA had comparable permeability (log *P*_E_ = − 6.6). The structural analysis by NMR suggested that a combination of factors might contribute to the observed permeability of the cyclo hexapeptide, including intramolecular hydrogen bonding, steric protection of amide NH groups from solvation, and the relative stability of impermeable open conformations in water. The same group further established an in silico prediction system for passive membrane permeability of cyclic peptides by calculating free energy of insertion (∆*G*_I_) into membrane (Rezai et al. 2006a). The results showed a strong correlation (*R*^2^ = 0.96) between ∆*G*_I_ and the permeability of cyclic peptides determined in PAMPA assay, supporting the hypothesis that internal hydrogen bonding is critical for passive membrane permeability.

## NPAAs and oral bioavailability

**Fig. 3:**
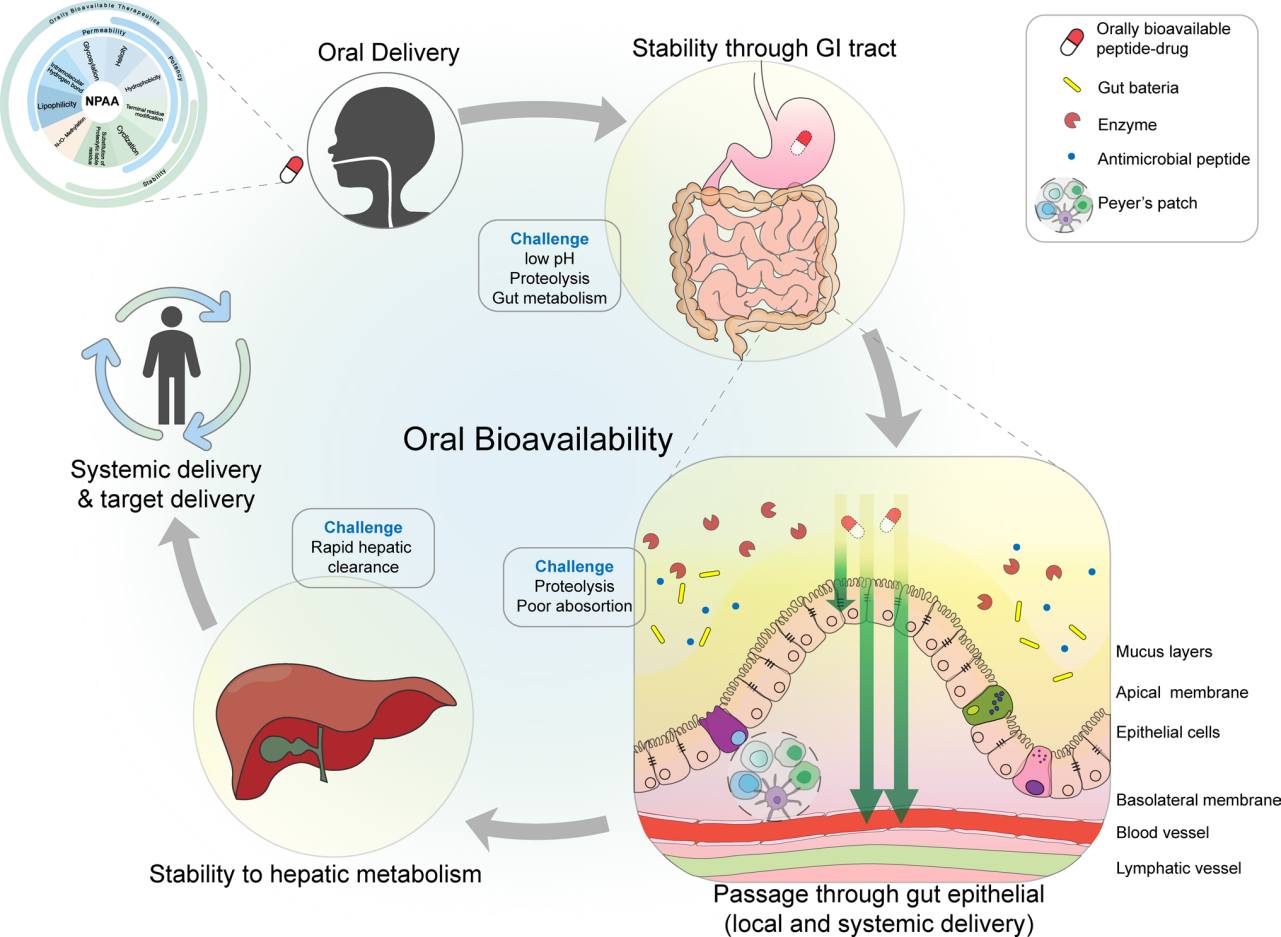
Schematic of orally bioavailable peptide therapeutics for local and systemic delivery. Non-proteinogenic amino acids are introduced in the peptide sequence to overcome challenges of oral delivery

Oral bioavailability (F) refers to the percentage of a drug that reaches blood circulation after it is orally administrated. Oral bioavailability of peptide therapeutics is determined by a combination of biophysical and chemical properties such as potency, solubility, proteolytic and metabolic stability, and permeability (Fig. 3). Lipinski’s rule of five describes the classic criteria to predict an orally bioavailable small molecule: H-bond donors ≤ 5, H-bond acceptors ≤ 10, molecular weight (MWT) ≤ 500, and calculated octanol–water partition coefficient (cLogP) < 5 (Lipinski et al. 2001). Veber revised the theory in 2002 and proposed only two criteria for high oral bioavailability: number of rotatable bonds ≤ 10 (preferably < 7); and polar surface area ≤ 140 Å^2^, or the total number of H-bond donors and acceptors ≤ 12 (Veber et al. 2002). Thus far, no guidelines have been established to describe the parameters that dictate oral peptide bioavailability. However, incorporation of NPAAs appears as a prerequisite to satisfy the requirements of peptide oral bioavailability.

LogP is a commonly used parameter for measuring molecular hydrophobicity/lipophilicity. It was previously reported that N- and O-methylations stabilize intramolecular hydrogen bonds. It also improves the lipophilicity by masking the ionizable acidic group, leading to the enhanced oral bioavailability (Ghose et al. 1998; Leach et al. 2006; Ritchie et al. 2015). For example, somatostatin cyclopeptidic analog cyclo (‐PF-(D-Trp)^8^K^9^TF^11^‐) had minimal oral bioavailability in rats (under detectable concentration in plasma), while its methylated analog (PF-(D-^Me^N-W)-(^Me^N-K^9^)T-(^Me^N-F^11^)) showed 10% oral bioavailability. A colorimetric assay revealed a robust increase of bilayer liposome interaction for this analog. However, the analog with N-methylation at Lys9 and Phe11 interacted with liposome less favorably than the other analogs with the same number of N-methylated residues (D-W^8^F^11^ and D-W^8^K^9^). This finding indicated that the position of N-methylation plays a key role in oral bioavailability when cLogP values are the same (Biron et al. 2008; Chatterjee et al. 2008). The cyclic hexapeptide cyclo (LLLLPY) mentioned earlier was shown not to be orally bioavailable. However, methylated (cyclo [L^Me^L^Me^LLP^Me^Y]) showed 28% bioavailability in rats (White et al. 2011). The investigational O-methylated drug, oprozomib (2-Me-5-thiazole-Ser(OMe)-Ser(OMe)-Phe-ketoepoxide), the next-generation analog of the tetrapeptide drug carfilzomib, is administrated intravenously. Oprozomib had an absolute bioavailability (*F*) of 17–39% in rodents and dogs and an equivalent antitumor activity in preclinical studies (Zhou et al. 2009) (ClinicalTrial.gov 2020). Cyclosporin A (CsA) is an FDA-approved immunosuppressant drug with 27% oral bioavailability in rats (White et al. 2011). CsA is composed of 11 amino acids, 7 of which are methylated (MeBmt-Abu-^Me^G-^Me^L-V-^Me^L-A-(D-Ala)-^Me^L–^Me^L-^Me^V) MeBmt: (4*R*)-4[(*E*)-2-butenyl]-4-[*N*-di-methyl-l-threonine]; Abu: l-aminobutyric acid) (Survase et al. 2011). Interestingly, CsA is thought to violate every aspect of Lipinski’s rule of five, as its molecular weight is way above 500 Da and possesses seven methyl groups serving as H-bond donor. The optimal pattern of N-methylation in CsA has resulted in favorable intramolecular hydrogen bonding and is considered as one big contributor to its oral bioavailability (White et al. 2011).

Increased lipophilicity of a peptide can be achieved by incorporating fatty acid-conjugated NPAAs or lipid-based formulation. PMX53 (^AC^F-Orn-P-^D^Cha-WR) is a cyclic peptide inhibitor of the anaphylatoxin receptor C5aR1. The lipophilic variant PMX205 (hydrocinnamate-Orn-P-^D^Cha-WR) improved its bioavailability from 8.6 to 22.6% in mice administered per-orally (Hawksworth et al. 2017; Kumar et al. 2020). Desmopressin (dDAVP, Mpa-(mercaptopropanoic acid)-YFQNCP(D-Arg)-G) is an antidiuretic hormone approved by FDA (Vorherr 2015). The oral bioavailability of desmopressin is limited (0.1% in human) due to its high hydrophilicity. However, medium-chain fatty acid-based formulation was used to overcome this problem. In a phase I study, desmopressin in medium-chain fatty acids formulation showed 2.4% oral bioavailability, while the non-formulated desmopressin was undetectable (Fjellestad-Paulsen et al. 1993; Leonard et al. 2006). In another study, F% in rats was increased by tenfold when desmopressin was delivered in monohexanoin with saline as the control vehicle. CAT, the lipophilic nonapeptide analog (Mpa-(D-Tyr(ethyl))-FVNCP(D-Arg)-G) showed a higher F% than desmopressin in a saline formulation. Interestingly, the lipid-based formulation did not benefit the oral bioavailability of CAT. This finding suggested that fatty acid side chains may play a similar role in oral bioavailability as the lipid-based formulation (Lundin et al. 1997). Semaglutide was formulated with a small fatty acid sodium *N*-[8-(2-hydroxybenzoyl) amino caprylate (SNAC) and is the first approved oral GLP-1 receptor agonist. SNAC is an absorption enhancer. It increases lipophilicity and enhances stomach absorption of the active peptide ingredient (Buckley et al. 2018; Li et al. 2018; Bucheit et al. 2020). Given the desmopressin and its analog as an example, it is possible that an SNAC-conjugated NPAA may also promote oral bioavailability of peptides such as semaglutide. Long-chain fatty acid (LCFA) conjugation can also increase oral bioavailability by binding to fatty acid transport protein 4 (FATP4, SLC27A4). Exendin-4 with LCFA conjugation at the C-terminal His and packaged in liposome showed 24.8% oral bioavailability in mice with efficient blood glucose regulation, while free exendin-4 was unable to show such impact (Hu et al. 2020).

Conformational change induced by NPAAs to enhance membrane permeability can be a key contributor of peptide oral bioavailability. It is more likely for macrocycles to be orally bioavailable due to the limited conformational constrain, smaller hydrodynamic radius, and the ability to form intramolecular hydrogen bonds. The cyclic CsA demonstrated excellent oral bioavailability without compromising bioactivity. NMR and X-ray studies revealed that the hydrophilic patches in CsA are exposed in an aqueous environment to allow cyclophilin binding. In a low polarity environment, a cis-amide bond is formed between *N*-methyl-Leu9 and *N*-methyl-Leu10 of CsA. As a result of hydrogen bond formation among the amide-hydrogens, *N*-methyl groups are turned outward. This new conformation facilitates passive membrane permeability and likely is the main contributor to the high oral bioavailability of CsA (Horst Kessler et al. 1990; Fesik et al. 1991; Altschuh et al. 1992; Bock et al. 2013). CsA portrays a “chameleon-like” behavior; it adopts different hydrogen bonding patterns in different environments to change its conformation and polarity. The “chameleon-like” behavior has gained much attention for describing the oral peptide drugs chemical space (Danelius et al. 2020). The native peptide α‐conotoxin Vc1.1 (G^1^C^2^C^3^SDPRC^8^NYDHPEIC^16^) contains two disulfide bonds between Cys2–Cys8 and Cys3–Cys16. The linear variant was cyclized by adding a C-terminal motif GGAAGG to link the C- and N-termini together. Both peptides showed analgesic functions by inhibiting GABA_B_‐modulated N‐type (CaV2.2) channel in vitro. The liner version demonstrated effective pain relief when it was administrated subcutaneously (Lam et al. 1991). The orally delivered cyclic α‐conotoxin Vc1.1 showed significant pain relief activity up to 4 h (Satkunanathan et al. 2005; Clark et al. 2010). Linaclotide (*F*% = 0.1%) and PTG 200 (Protagonist Therapeutics, clinical trial phase II) are among the orally administered macrocyclic peptides for local targeting of gut restricted diseases (Bryant et al. 2010).

Oral bioavailability of a peptide can be affected by numerous factors. NPAAs play a key role in oral bioavailability of a few peptides discussed here. However, the underlying principles of what governs oral bioavailability is not yet known. The Arg-vasopressin analog desmopressin with the l-Arg to d-Arg replacement resulted in improved potency, reduced renal clearance, and prolonged plasma half-life (55 min compared to 5 min in vasopressin) (Rado et al. 1976; Vilhardt and Lundin 1986; Vilhardt et al. 1986). The macrocycle ulimorelin (TZP-101) is a phase III ghrelin growth hormone agonist. It contains d-Phe and one N-methylation site and showed an oral bioavailability of 24% in rats and monkeys (Hoveyda et al. 2011). It seems that the use of d-amino acid is prominent in oral peptides, possibly to reduce proteolysis and promote conformational constraints. Although oral delivery of polypeptides seems feasible on a case-by-case basis, the use of d-amino acid, N-methylation, macrocyclization, and reduction of size appear to be the right steps to improve oral bioavailability. Properties that derive peptide oral bioavailability and some examples are described in Table 1.

**Table 1:** Properties that derive peptide oral bioavailability and some peptide examples

Peptide property improvement	Peptide example before modification	Non-proteinogenic amino acid introduced modification
Stability	Angiogenic heptapeptide (DRVYIHP)	Substitution of proteolytic liable residues
	MP (KKVVFKVKFKK)	
	GLP-1 (HAEGTFTSDVSSYLEGQAAKEFIAWLVKGR)	Substitution of proteolytic liable residues; side chain modification for half-life extension
	GLP-2 (HADGSFSDEMNTILDNLAARDFINWLIQTKITD)	Substitution of proteolytic liable residues; terminal residue modification
	Anti-angiogenic peptide F56 (WHSDMEWWYLLG)	Terminal residue modification for half-life extension
	YIK (EMTWEEWEKKIEEYIKKIEEILKKSQNQQLDL)	
	279–287 sequence of glycoprotein-D (LLEDPVGTVA) of herpes simplex virus (HSV)	Cyclization
	LP1 (Ac-L(Ant)FFK-CONH_2_)	
Potency	Compstatin (ICVVQDWGHHRCT)	Increase of hydrophobicity and polarity
	HLA-DQ blocking peptide (ADAYDYESEELFAA)	Increase of hydrophobicity; NPAA incorporation for increasing affinity
	12p1 (RINNIPWSEAMM)	
	GLP-1 (HAEGTFTSDVSSYLEGQAAKEFIAWLVKGR)	NPAA incorporation for increasing affinity
	GLP-1 (HAEGTFTSDVSSYLEGQAAKEFIAWLVKGR) and glucagon (HSQGTFTSDYSKYLDSRRAQDFVQWLMNT)	Introduction of dual-agonism, stabilization of peptide helicity
	Hemagglutinin binder (ARLPRTMVHPKPAQP)	Increase of hydrophobicity
	YIK (EMTWEEWEKKIEEYIKKIEEILKKSQNQQLDL)	
	C34 (WMEWDREINNYTSLIHSLIEESQNQQEKNEQELLGSGC)	
Permeability	AMP (Ac-KA∆FWK∆FVK∆FVK-CONH_2_)	Enhancement of peptide helicity
	Cbf-14 (RLLRKFFRKLKKSV)	
	Octa-arginine analogs (RRRRRRRR)	
	BID BH3 peptide (EDIIRNIARHLAQVGDS-Nle-DRSIW)	Enhancement of peptide helicity and lipophilicity
	Bim BH3 peptide (IWIAQELRRIGDEFNAYYARR)	Enhancement of peptide helicity
	BBB shuttles (N-MePhe)_n_	Enhancement of hydrophobicity and lipophilicity
	Leu-Enk (YGGFL)	Enhancement of lipophilicity; terminal residue modification; glycosylation
	Met-Enk (YGGFM)	Glycosylation
	EM-1 (YPWF)	Terminal residue modification; enhancement of hydrophobicity and lipophilicity; glycosylation
	Cyclo (LLLLPY)	Cyclization
Oral bioavailability	Somatostatin analog cyclo (-PF-(D-Trp)^8^K^9^TF^11^-)	Formation of intermolecular hydrogen bonds by cyclization
	Cyclo (LLLLPY)	
	Tetrapeptide drug carfilzomib	
	PMX53 (^AC^F-Orn-P-^D^Cha-WR)	Enhancement of lipophilicity
	Desmopressin (dDAVP, Mpa-(mercaptopropanoic acid)-YFQNCP(D-Arg)-G)	Enhancement of lipophilicity Enhancement of lipophilicity; side-chain modification for absorption enhancement
	Semaglutide (H-Aib-EGTFTSDVSSYLEGQAAKEFIAWLVRGRG)	
	α-Conotoxin Vc1.1 (G^1^C^2^C^3^SDPRC^8^NYDHPEIC^16^)	Cyclization
	Ulimorelin (TZP-101)	Cyclization; conformational change

### Utilizing NPAAs in selections

**Fig. 4:**
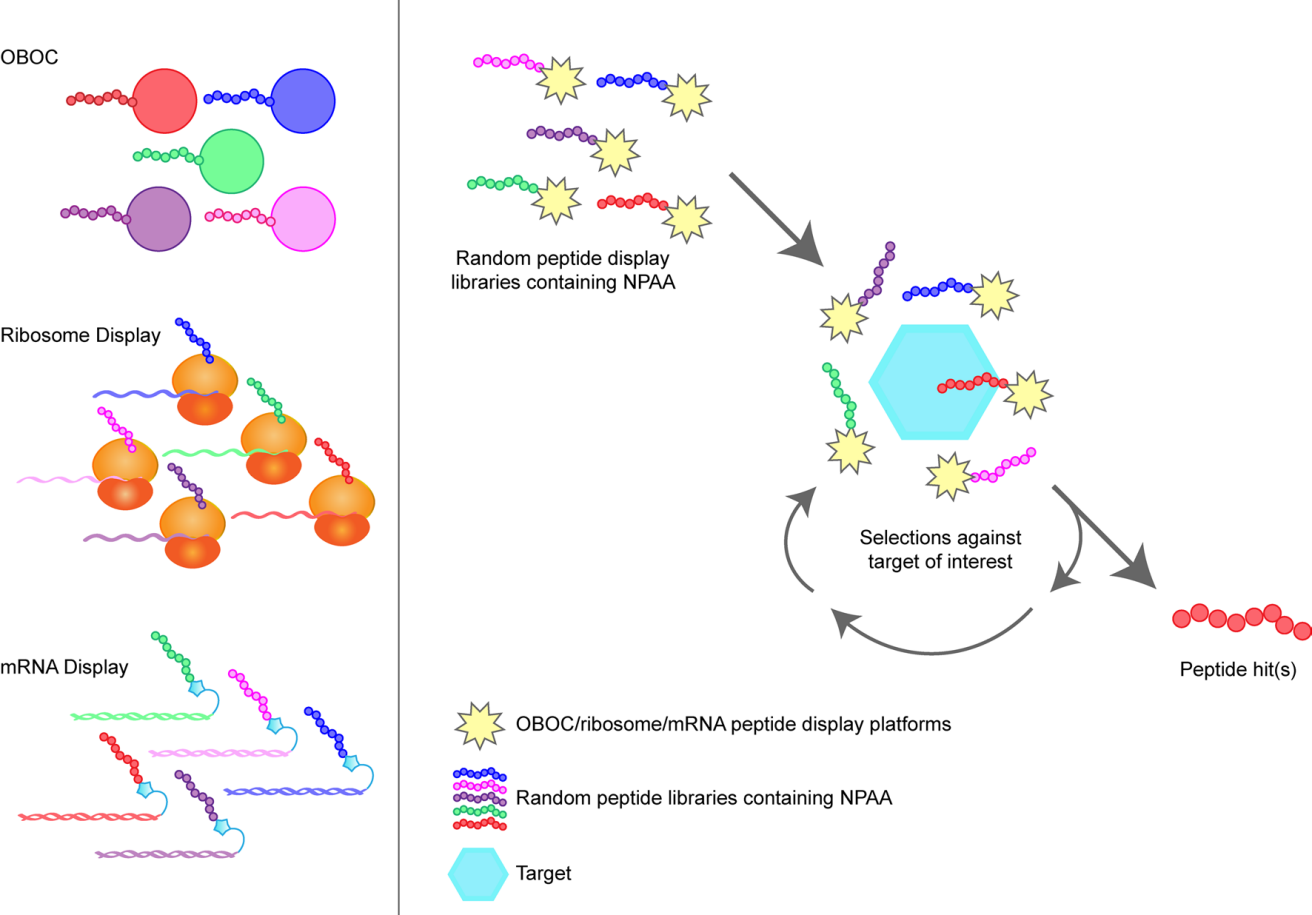
Incorporation and utilization of NPAAs in peptide display platforms. NPAAs can be incorporated in different peptide display platforms such as OBOC, ribosome display, and mRNA display. These random peptide libraries can be used for affinity selection to identify hit candidates already containing NPAAs. Hit candidates can be improved for stability, potency, permeability, and oral bioavailability

Novel peptide discovery engines have enabled utilization of NPAA in peptide sequence, allowing discovery of de novo peptides with the desired properties. Peptide discovery platforms such as one-bead-one-compound (OBOC) (Lam et al. 1991), ribosome display (Watts and Forster 2012), and variations of mRNA display (amber suppressor tRNA (Noren et al. 1989) or Random nonstandard Peptide Integrated Discovery (RaPID) platforms (Passioura and Suga 2017) allow incorporation of NPAA in peptide sequence (Fig. 4). As a result, highly diverse peptide libraries (Passioura and Suga 2017) can be constructed and selected to identify target-specific peptides.

In the OBOC combinatorial library method, display of numerous copies of the same peptide on a single bead is made possible by a “split-mix” synthesis approach (Lam et al. 1991). NPAA can be incorporated during the synthesis around a natural amino acid scaffold (Aina et al. 2005; Goksel et al. 2011; Raghuwanshi et al. 2017). A single library containing tens of thousands to millions of peptides can be selected against the target of interest to identify hits. An example of a peptidomimetic that has emerged from this platform to phase I clinical trials is a single digit picomolar peptidomimetic ligand (LLP2A) against α4β1 integrin (Peng et al. 2006). LLP2A is used to image α4β1-expressing lymphomas with high sensitivity when conjugated to a fluorescent dye.

In ribosome display, ribosomal machinery is used to generate a peptide library based on mRNA encoding region. Transcription/translation machinery can be supplied either through cell extracts or by cell-free system containing all the required components for translation, also known as Protein synthesis Using Recombinant Elements or PURE (Shimizu et al. 2001). The translated protein remains tethered to the stalled ribosome and its encoding mRNA due to the lack of stop codons and release factors (RF). This highly diverse (> 10^12^) non-covalent ternary complex (mRNA, peptide library and ribosome) library is selected against the target molecule (Lipovsek and Pluckthun 2004; Watts and Forster 2012). Early incorporation of NPAA in ribosome display involved the chemical acylation of NPAA to amber suppressor tRNA (CUA), and later to opal (UCA) and ochre tRNAs (UUA) (Noren et al. 1989). The codons for the different suppressors were incorporated into the mRNA code, allowing for incorporation of the NPAAs such as biotinylated-Met (Watts and Forster 2012), biocytin (Li et al. 2002) and *N*-methyl amino acids (Subtelny et al. 2008). Incorporation of these NPAAs have demonstrated that the ribosome can translate building blocks beyond the 20 natural amino acids; however, the number of NPAA that can be included in a peptide is limited to the number of suppressor codons. mRNA display also utilizes ribosomal translation machinery to translate proteins in vitro based on the mRNA genetic coding region. Unlike ribosome display, the translated protein is covalently attached to the mRNA through a puromycin moiety to provide a physical linkage between peptide genotype to phenotype (Roberts and Szostak 1997). This highly diverse (> 10^12^) mRNA peptide library complex can be selected against the target, and their amino acid sequence is determined by next-generation sequencing. In RaPID platform (PeptiDream Inc.), mRNA display is combined with a novel family of ribozymes, called flexizyme (Murakami et al. 2006). Flexizymes can charge any tRNA with almost any NPAA. As a result, highly diverse NPAAs are incorporated in the peptide sequence. RaPID has been used to identify macrocyclic peptides containing NPAA with high affinity for several targets (Yamagishi et al. 2011; Hayashi et al. 2012; Tanaka et al. 2013). Currently, a few candidates discovered from mRNA display platform are in the early stages of clinical trials (Huang et al. 2019). An example is the anti-PDL-1 peptide (PeptiDream–BMS) in phase I clinical trials, but with limited public information. Zilucoplan (RA101495) is a 15-amino acid macrocyclic peptide discovered from mRNA display platform by RA pharmaceuticals that specifically binds to complement component 5 with sub-nanomolar affinity. The peptide is being investigated in phase II–III trials for tissue-based complement-mediated disorders such as generalized myasthenia gravis, immune-mediated necrotizing myopathy, and amyotrophic lateral sclerosis (Howard et al. 2020).

### Immunogenicity risks due to incorporation of NPAAs

Incidences of immunogenicity are reported for 89% of therapeutics, of which half were observed to affect the efficacy of the drug (Schultz et al. 2018). Immunogenicity is triggered when CD4 T-cells recognize peptide fragments presented by antigen-presenting cells (APC). As a result, production of neutralizing and non-neutralizing antibodies, formation of immune complexes, complement and mast cell activation, inflammation, and anaphylaxis can ensue (Jawa et al. 2013). Therapeutics with higher structural similarity to endogenous proteins/peptides have relatively lower immunogenicity risk. Exenatide, a GLP-1 analog derived from Gila monster, shares 53% sequence identity to human GLP-1. Patients treated with exenatide have shown higher incidence of anti-drug-antibody (ADA) compared to patients treated with liraglutide (97% sequence identity to human native hormone) (Buse et al. 2011).

In some cases, NPAA incorporation has resulted in reduced immunogenicity. Efpeglenatide is a modified analog of exendin with N-terminal His replaced with 4-imidazoacetyl group. A flexible PEG linker conjugated to Lys27 was used to facilitate its coupling to Fc fragment of IgG4. It was shown that efpeglenatide was much less immunogenic and had lower incidence of treatment-emergent ADAs. Interestingly, no incident of neutralizing antibodies was reported (Rosenstock et al. 2019). Two BBB peptide shuttles H_2_N-HAIYPRH-CONH_2_ (HAI) and H2N-THRPPMWSPVWP-CONH214 (THR) were altered to the corresponding retro-D-peptide. Immune response (IR) was not detected in mice by retro-D-HAI or retro-D-THR, whereas a moderate immunogenicity was observed with the parental peptides. The authors suggested that both d- and l-forms might be recognized by B cell receptor. However, the retro-D version is presented by APC less efficiently, since it is resistant to proteolysis. This “immunosilencing” behavior was reversed when the peptide was conjugated to Keyhole limpet hemocyanin. The complex elicited IR in rabbit, suggesting that retro-D class of peptides can also be used as vaccines (Arranz-Gibert et al. 2018). Substitution of the residues that interact with HLA or T-cell receptors with NPAAs may reduce the risk of immunogenicity (Meister et al. 2019). The peptide segment corresponding to residues 35–55 (MEVGWYRSPFSRVVHLYRNGK) of myelin oligodendrocyte glycoprotein (MOG) was considered as a potential peptide vaccine for multiple sclerosis. The residue Phe44 was identified as the key T-cell receptor binding spot. Replacing Phe44 in MOG 35–55 with ^β^Phe attenuated T-cell autoreactivity in mice, indicating the beneficial role of β-amino acid in reducing immunogenicity (McDonald et al. 2014). Alternatively, specific NPAA incorporation is shown to enhance immunogenicity. Grunewald and coworkers incorporated highly immunogenic non-proteinogenic amino acid *p*-nitrophenyl-alanine (pNO2Phe)- on the surface residue (Tyr86) of murine tumor necrosis factor α (m-TNF-α). The new analog resulted in T-cell-dependent high titer neutralizing antibody response that was cross reactive to WT m-TNF-α. This resulted in an efficient protection in mice against lipopolysaccharide (LPS) challenge. Similarly, immunization with a pNOPhe mutant (Tyr43 and Tyr108) of murine retinol binding protein (RBP4) elicited high titer IgG antibody response against wild-type mRBP4. This suggests that incorporation of certain NPAA might result in immunogenicity and breaking immune tolerance against cancer-associated antigens (Grunewald et al. 2009).

## Conclusion

NPAAs provide a much more diverse set of building blocks for improving peptide pharmacokinetic properties. As illustrated in this review article, the half-life, specificity, potency, membrane integration, and conformation of peptides can be optimized by use of NPAAs. A balanced combination of these attributes can lead to peptide bioavailability. Although the systemic uptake of orally delivered peptides can be affected by various factors and the classic Lipinski’s and Veber’s rules are not valid in the oral peptide space, NPAAs have shown to provide a powerful toolbox for the rational and empirical design of oral peptides.

## Abbreviations

A: Alanine
AA: Amino acid
Aβ: Amyloid-β
ACE: Angiotensin-converting enzyme
AD: Alzheimer’s disease
BBB: Blood–brain barrier
C: Cysteine
Cha: Cyclohexylalanine
CNS: Central nervous system
D: Aspartic acid
DPP-3: Dipeptidyl peptidase 3
DPP-4: Dipeptidyl peptidase 4
E: Glutamic acid
F: Phenylalanine
G: Glycine
GLP-1: Glucagon-like peptide
H: Histidine
HIV-1: Human immunodeficiency virus-1
HLA: Human leukocyte antigen
I: Isoleucine
K: Lysine
M: Methionine
N: Asparagine
NAA: Natural amino acid
NRP: Non-ribosomal peptide
Orn: Ornithine
P: Proline
PAMPA: Parallel artificial membrane permeability assay
PEG: Polyethylene glycol chain
PTM: Post-translation modification
Q: Glutamine
R: Arginine
S: Serine
T: Threonine
NPAA: Non-proteinogenic amino acid
V: Valine
W: Tryptophan
Y: Tyrosine
